# A Case of Paraplegia from Peripheral Irritation

**Published:** 1874-04

**Authors:** G. W. Stewart

**Affiliations:** Muscatine, Iowa


ARTICLE III.—*A Case of Paraplegia, from Peripheral Irritation.* By G. W. STEWART, M.D., Muscatine, Iowa.

In May, 1872, while serving as county physician, I was called to the county poor house to see an inmate, Maggie O——, aged 18, who complained that she could not pass her water.

I introduced a catheter, and evacuated the bladder of its contents, only about four ounces of very highly colored urine. Upon inquiry, I learned that a larger quantity had not been passed on any one day for a period of a month, and on some days the quantity was even less.

She complained of some pain, though not intense, over the region of the kidneys, and since the inability to pass her water had existed, (a period of twenty-four hours), there was in the neigborhood of the bladder a burning or tingling sensation, more or less constant, and most marked when she attempted to pass her water.

Upon inquiry, I learned that she never had been robust, and discovered that she was of a scrofulous diathesis, having had, in fact, a few years previous, a scrofulous ulcer in the glands of the neck, the characteristic cicatrix of which, with puckered tissues bordering the same, being particularly noticeable.

Continuing my examination, I subjected the urine to the heat and nitric acid tests for Bright's disease; examined it with the microscope, and tested its specific gravity. Chemical tests revealed little (*i. e.*, tests tried) as to the real character of its sediments observed, and its high color, showing neither albuminuria, nor a diminution of urea; but the microscope presented to view blood corpuscles, and casts cylindrical in form, and evidently from the convoluted tubes. Specific gravity normal, 1020.

Subsequent to this, I repeated frequently the same tests, and several times with like results. However, at a later period, the specific gravity was lower.

TREATMENT.

Not recognizing, for some time, any connection or relation existing between the paralysis of the bladder, and partial paralysis of the sphincter ani and muscles of lower extremities which followed, on the one hand, and that of the desquamative nephritis on the other, I prescribed as though they were separate affections; administering, for the first, strychnia, beginning with $\frac{1}{60}$ of a grain, three times daily, and increasing gradually to $\frac{1}{8}$ of a grain; for the second, I endeavored to relieve the kidneys from active duty by stimulating the skin and bowels to eliminate in an increased degree fluids from the body, administering, for the purpose, diaphoretics and cathartics, using of the former, nitrate of potash and Dover's powders, the latter of which served also to allay pain and inflammation in the kidneys. As cathartics, I used the salines; at the same time directing that the patient be kept unexposed to cold or moisture, and that she take at intervals of from two to three days a hot air bath; her diet to consist of milk and broths.

This course of treatment I followed for a period of six weeks, the first two of which saw in our patient some improvement, the quantity of urine being greater, of a better color and general appearance; and the bladder responded to the stimulation it received—as I at that time thought through the influence of the strychnia—and voided urine for a few days quite naturally. But after a few days, seeming to have become acclimated, so to speak, to the remedial agents used, the case changed fronts, not only ceasing to improve, but became worse; the quantity of urine passed diminishing, and the paralysis intensifying and extending to other parts than that hitherto affected, when we had a completely paralyzed bladder, sphincter ani and muscles of the lower extremities partially so.

Our patient having become by this time quite considerably debilitated by the action of cathartics and the disease combined, I dropped, for a time, the former, and administered in their stead, tonics. And having given, as I thought, the strychnia a fair trial, with no apparent benefit, I dropped it, and took up electricity, administering it first directly to the bladder by placing one pole of the battery in the stream of urine as it was voided through a catheter, making it (the urine) conduct the current directly to the bladder, and the other pole, placed over the spinal column, completed the circuit.

This operation was performed daily for another six weeks, with no apparent change, except, now and then, some dribbling of urine. There being no improvement, I did not feel much encouraged, and my patient was still less so, and refused to do anything further in the way of treatment.

At about this time, I read before our County Medical Society a history of her case, in which I expressed surprise that uræmia had not manifested itself, considering the long duration of the disease, and the limited amount of urine passed (about four ounces per day); expressing the opinion that it would seem as though she must be a malingerer, at least so far as the quantity of urine voided was concerned; that it would seem impossible for so small an amount of urine to be passed for a period of three months without uræmia declaring itself; yet, having faith in the moral integrity of my patient, I could hardly believe her to be a malingerer. However, I directed that she be closely watched, which was done, I thin k, faithfully by a person of undoubted integrity, but she could not be caught practicing or attempting to practice deception.

Never having seen laid down in the books any method by which one could determine, independent of the testimony of a patient, whether or not a bladder was paralyzed, or whether an inability on the part of the patient to urinate really existed, I was at a loss how to proceed further, but being frequently at the poor house, I one day introduced into my patient's bladder a male catheter, having the instrument curved to about the extent it would be when used in a male. I then attempted to rotate the instrument, which I found could be done, in which case the inner extremity would be made to describe a circle. To do this with facility, as in the present instance, the bladder must needs be in a state of at least partial distension; and, being able to rotate the instrument as well with the bladder empty as when it contained urine, I concluded that the organ was paralyzed, and that I had discovered a method of diagnosis, new, at least to me, by which I could determine in such cases whether a bladder were or were not paralyzed. I have since, on a number of occasions, tried the plan, and it has operated, as at first, to my satisfaction.

In the meantime, the case being left to itself, uræmia speedily declared itself, and the paraplegia was intensified; vertigo, dimness of vision at times, and irritability of the stomach being present, accompanied by the almost complete loss of power in muscles of lower extremities.

At this juncture, our patient wished me again to take her in charge, which I did, though with little hope of doing her any permanent good.

I put her upon diaphoretics and cathartics, using elaterium; tried again strychina, and followed the same hygienic and dietetic treatment adopted at first.

Upon this line of treatment our patient improved from the first, and, at the end of two months, there being an absence of the more marked symptoms of uræmia, the kidneys acting with a considerable degree of vigor, with a complete absence of all paraplegic symptoms, our patient thought sufficient had been accomplished, and desired no more treatment, and, accordingly, she received none.

After the lapse of two months, however, there was a return of all the old symptoms of pain over the kidneys, a burning or tingling sensation of the bladder, scant and highly colored urine, containing blood corpuscles and casts, with paralysis of the bladder. I pursued the same course of treatment for the kidneys as before; but noticing that throughout the history of the case the two conditions of nephritis and paraplegia moved together; that, where the former attained its acme the latter did also; likewise, when there was an amelioration in the symptoms of the former, there was also an amelioration in those of the latter, I came to the conclusion that a relation existed between them, and that the latter was produced by the former; therefore, I left the paraplegia entirely to itself. I believed that the paraplegic condition was due to peripheral irritation, set up by the diseased kidneys by or through reflex action of the spinal cord; and that the requirements were to direct my treatment principally to the kidneys.

This I did, ignoring altogether at first the paraplegia, and the case began to improve, and continued to do so, as it had at a previous time, until there was an absence of the more marked symptoms of uræmia, an increase in the amount of urine, an improvement in general appearance of the secretion, which showed again a specific gravity but little below healthy, a marked improvement in that particular; also, the paraplegic condition showed a marked improvement, the bladder voiding urine quite naturally but not perfectly, with the sphincter ani and muscles of lower extremities responding to stimuli, and acting with their wonted vigor.

Here the case seemed to halt, and, a little out of curiosity, I tried again by turns, with no other treatment, strychnia and electricity; but no improvement by that means could be brought about in the action of the bladder, and I became more than ever convinced, that until a cure could be found for uræmia, the bladder, in this case, could not be made to act in a degree perfectly normal.

The case has passed out of my hands, but remains about as I left it a year since.

